# Glycopyrrolate in comparison to hyoscine hydrobromide and placebo in the treatment of hypersalivation induced by clozapine (GOTHIC1): a feasibility study

**DOI:** 10.1186/s40814-019-0462-1

**Published:** 2019-06-17

**Authors:** Inti Qurashi, Simon Chu, Richard Drake, Victoria Hartley, Imran Chaudhry, J. F. W. Deakin, Nusrat Husain

**Affiliations:** 1 Ashworth Hospital, Mersey Care NHS Foundation Trust, Maghull, UK; 2 Faculty of Medicine, Biology and Health, University of Manchester, Manchester, UK; 3 Ashworth Research Centre, Mersey Care NHS Foundation Trust, Maghull, UK; 4 School of Psychology, University of Central Lancashire, Preston, UK

**Keywords:** Clozapine-induced hypersalivation, Drooling, Glycopyrrolate, Hyoscine, Feasibility study, Refractory schizophrenia, Side effects, Cognition

## Abstract

**Background:**

Clozapine-induced hypersalivation (CIH) is a common side effect of clozapine treatment and is disliked by clozapine patients, potentially threatening adherence to clozapine treatment. We proposed a trial of alternative medications, hyoscine and glycopyrrolate, for the treatment of CIH and the primary objective of the feasibility study was to assess the recruitment and retention of community clozapine patients as well as assess the metrics of the primary hypersalivation measure.

**Methods:**

This 11-month trial took place in two NHS trusts in northwest UK. Participants were community-dwelling clozapine patients aged 18–65 years who were suffering from CIH, and were recruited from community mental health clinics. They were randomised using a telephone randomisation service to receive either hyoscine (1 week at 0.6 mg daily, 3 weeks at 0.9 mg daily), glycopyrrolate (1 week at 2 mg daily, 3 weeks at 3 mg daily) or placebo. Participants and investigators were blinded to which study arm the participants had been randomised to. We collected data on salivation levels and side effects on a weekly basis and also assessed cognition at the beginning and end of the trial. We also interviewed a sample of participants after the trial to gather information on their experience of having taken part.

**Results:**

One hundred and thirty-eight potential participants agreed to being contacted by researchers about participation in the trial and of these, 29 participants were randomised. Of these, four participants exited the trial before taking any trial medication, and two participants left the study owing to concerns of side effects. Data from four participants was missing, and complete data was available for 19 participants who completed the trial. The mean recruitment rate overall was 1.3 participants per site per month, and the overall retention rate was 76%. Interview data suggested that participants’ experiences of trial participation were overwhelmingly positive.

**Conclusions:**

The feasibility study demonstrated that a trial of alternative medications in the treatment of CIH is feasible; patients were willing to be randomised to the trial and retention rate was high.

**Trial registration:**

ClinicalTrials.gov, NCT02613494, registered 24 November 2015

## Background

Clozapine is the only medication licenced for the treatment of resistant schizophrenia and in almost all cases is a lifelong prescription [1]. Clozapine-induced hypersalivation (CIH) is a common side effect of clozapine treatment, occurring at any stage of treatment in greater than 90% of patients [2]. CIH carries with it a profound social stigma, lowering self-esteem, increasing social isolation and exacerbating psychological problems [3]. It can cause inflammation of the salivary glands and surrounding skin infections [4] and affect sleep quality [3]. A survey of patients prescribed clozapine found that hypersalivation was the most unacceptable side effect of clozapine treatment and crucially, the burden of adverse side effects may eventually lead to patients discontinuing clozapine treatment [5]. Therefore, effective treatment of CIH is vital to patient experience and wellbeing.

In the UK, the most commonly used medication for CIH is hyoscine hydrobromide (‘hyoscine’), an anti-muscarinic licenced as a motion sickness prophylactic and as a pre-operative drug to dry secretions. This is partly because CIH is listed as an unlicensed use for hyoscine in the British National Formulary [6]. However, there is no convincing evidence for any drug as an effective treatment for CIH [7–9] although one recent small trial has shown encouraging results with hyoscine [10]. Additionally, a wide range of side effects has also been linked to hyoscine [11, 12], most commonly drowsiness, dizziness and constipation (which can be a lethal side effect of clozapine treatment). Because it crosses the blood-brain barrier, hyoscine may also cause cognitive deficits, including impairments of visual and verbal memory [13]. This is significant because cognitive deficits in schizophrenia are common and associated with poor long-term outcomes [14].

Glycopyrronium bromide (‘glycopyrrolate’) is an anti-muscarinic with poor blood-brain barrier penetration [15]. It is widely used in the UK as a pre-anaesthetic agent because of its long-lasting ability to decrease salivary production and gastric acid, and it is commonly prescribed by paediatricians in the treatment of drooling in children with neuro-developmental disorders (e.g. cerebral palsy). A small (*n* = 13) double-blind, randomised crossover study of glycopyrrolate and biperiden (a centrally acting anticholinergic similar to hyoscine) in CIH reported reduced hypersalivation scores in patients receiving glycopyrrolate [16]. Importantly, cognitive functioning assessment scores showed a significant reduction for participants receiving biperiden but not for glycopyrrolate. The authors concluded that glycopyrrolate could be a valid treatment for CIH although larger trials were required to confirm the finding. A Cochrane review [7] of the evidence base for the efficacy of pharmacological treatments for CIH concluded ‘there are currently insufficient data to confidently inform clinical practice, limitations of these studies are plentiful and the risk of bias high’ and ‘it seems reasonable to trial safe interventions for which there is a rationale’.

A large RCT is therefore required to evaluate the efficacy of hyoscine and glycopyrrolate, in comparison to placebo, in the treatment of CIH. However, such a trial presents a number of challenges including whether potential participants with CIH would agree to be randomised to a placebo arm, whether the proposed trial design and schedule of assessments would be acceptable to potential participants and establishing the metrics of the primary outcome measure to measure drooling. Therefore we conducted a feasibility study as a step towards a future large RCT. The feasibility study aims were to do the following:

1. Ascertain whether the proposed study design is acceptable to participants, including randomisation and use of telephone interviews to obtain data.
2. Ascertain whether the study interventions are acceptable to participants and indicate attrition rates and tolerability.
3. Assess the standard deviation of the proposed primary outcome measure, the Drooling Rating Scale, to inform a sample size calculation for a future efficacy RCT.

## Methods

The reporting of the study follows the CONSORT statement recommendations [17].

### Design

This 11-month feasibility study was a multi-centre, double-blind, randomised placebo-controlled trial of two investigational medicinal products (IMPs), hyoscine and glycopyrrolate, in the treatment of male and female patients with clozapine-induced hypersalivation.

Patients were closely involved throughout the development and design of the study. The initial research question was inspired by views of the side effects of clozapine treatment gathered from our patients [5], and the design of the study benefited from the views of service users who advised on the acceptability of the proposed recruitment strategy and trial procedures. A service user researcher also joined the trial management group to assist in overseeing the study.

Patients were randomised in a 1:1:1 ratio to one of three treatment arms, hyoscine, glycopyrrolate, or placebo. Participants who were already taking medication for hypersalivation when joining the study had that medication stopped for 1 week (the ‘washout’ period) prior to taking the IMP. Baseline measures of cognition, hypersalivation and side effects were measured prior to taking the IMP. In week 1 of the intervention, participants were administered a lower dose of IMP (0.3 mg hyoscine twice daily or 1 mg of glycopyrrolate twice daily). In weeks 2–4 of the intervention, participants were administered a full dose of IMP (0.3 mg hyoscine three times daily, or 1 mg of glycopyrrolate three times daily). Hypersalivation and side effects were measured at the end of weeks 1–4, and cognition was measured again at the end of week 4. A 4-week intervention is consistent with other clozapine-induced hypersalivation trials [10, 17] to allow for estimation of attrition rate.

### Recruitment

The target number of patients with complete data at baseline and follow-up in each of the three study arms was 14, and 42 in total, from three study sites. The sample size was determined by a number of factors. Forty-two would allow an estimate of the metrics for the primary outcome measure (Drooling Rating Scale) [18] and also allow an assessment of the recruitment and attrition rate, easily accommodating the 12-per-group rule of thumb recommended by Julious [19]. Both of these factors are feasibility aims. The predicted attrition rate was estimated at 20% using a conservative approximation based on previous studies using similar populations [10, 16]. Twenty percent attrition allows clear differentiation from the progression criterion (see below; 40% attrition), with > 80% power to detect a difference of this size with alpha 0.25 (1 tailed). This is consistent with the relaxed power and alpha criteria suitable for an early phase trial of this type [19, 20], specifically the alpha 0.25 criterion advocated by Schoenfeld [21].

Recruitment commenced in January 2017. Participants were included in the study if they were English-speaking, community-dwelling, between the ages of 18 and 65 years, prescribed clozapine for a minimum of 3 months, and experiencing hypersalivation. They were excluded if they had any medical conditions that affected hypersalivation (e.g. idiopathic Parkinson’s disease), neurological conditions that affected cognitive functioning, history of allergic reactions, contraindications or cautions to hyoscine or glycopyrrolate; had been prescribed other medications with a significant anticholinergic profile; or had been experiencing suicidal ideation.

Patients attending their regular community clozapine clinic appointment were approached by the clinic team about possible participation in the study. Once consent to contact was obtained, the research team contacted the patient to provide written information about the study, followed-up with a telephone call at least 2 days later and formal consent was taken by a researcher who visited the patient’s home. Occasionally, a researcher was present in the clozapine clinic when the patient was first approached by clinic staff and if the patient expressed interest in the trial, they were introduced to the researcher who was then able to explain the trial in more detail and provide written information. Under these circumstances, the researcher still followed-up by telephone at least 2 days later before arranging formal consent. Following formal consent, the researcher accessed the patient’s medication details from their GP to confirm eligibility. The Chief Investigator formally confirmed eligibility before patients were randomised.

Midway through the study, clinic staff suggested that patients might be more receptive to participation if they were already aware of the existence of the study prior to being approached about it by staff. In response to this, posters advertising a research study on medication to treat clozapine-induced hypersalivation were placed in clinic waiting areas to raise awareness. Clinic staff subsequently reported anecdotally that potential participants appeared more amenable to hearing more about the study when they were approached.

### Randomisation procedure

Randomisation services were provided by the Manchester Academic Health Sciences Centre – Clinical Trials Unit (MAHSC-CTU). The trial was blinded with three arms to be allocated in a 1:1:1 ratio. A computer generated list was drawn up by a statistician at MAHSC-CTU for 42 allocations and supplied to the trial pharmaceutical company to facilitate preparation and labelling of the bottles.

Once eligibility was confirmed by the Chief Investigator, the patient was randomised to one of the treatment arms. Third-party randomisation took place by telephone call to MAHSC-CTU. The researcher provided the participant’s initials, gender and date of birth, and the randomisation service returned a participant ID number and bottle number. The bottle number corresponded to numbered sets of IMP held by the trial pharmacy. The participant’s ID number was recorded in the case report form and on all study documentation related to that participant.

### Medication and blinding

IMPs and placebo were overencapsulated by the trial pharmaceutical manufacturer (Catalent UK) to look identical. Medication was bottled in study kits comprising one Week 1 bottle (containing 14 capsules) and three other bottles (each containing 21 capsules) for Weeks 2–4. The manufacturer labelled all medication bottles with bottle numbers under instruction from MAHSC-CTU who kept the list of bottle numbers and corresponding medication contents. MAHSC-CTU provided the trial pharmacy with a sealed list of bottle numbers and contents in the event that emergency unblinding was required.

Neither the participants nor the research team was aware of the trial arm to which any participant had been assigned. This was revealed to the study team by MAHSC-CTU only at the end of the trial. Following the end of the trial, the study team wrote to all participants to relay this information and thank them for their help in participating.

### Study visits and data collection

Formal consent and data collection was completed during visits to the participants’ homes or occasionally at a suitable alternative location (e.g. community clinic) if participants preferred not to have visitors at home. There were four visits (consent, baseline, maintenance, final) in total, with two additional visits (medication collection, exit interview) if optionally required.

In the consent visit, participants who had verbally agreed to take part in the study were visited by a researcher to answer any questions and clarify any study participation issues. At this visit, participants signed the consent form. Subsequent to this, further eligibility checks were completed using the participant’s medical history and current medications list, and when the Chief Investigator confirmed eligibility, the participant was randomised to the study. During the baseline visit at the beginning of Week 1, participants completed measures of cognition, salivation and side effects and were given a 1-week supply of IMP. During the maintenance visit (at the end of Week 1), participants were visited to complete the salivation and side effects measures and were also given a 3-week supply of IMP. At the end of Week 2 and Week 3, participants were telephoned to complete the salivation and side effects measures. In the final visit at the end of Week 4, participants completed measures of cognition, salivation and side effects.

Two other optional visits were possible. If prior to joining the study, the participant had been taking medication for salivation, this was removed from them during a medication visit that took place 1 week prior to the baseline visit. Also, at the end of the study, participants were given the option of taking part in an exit interview that would be conducted by an expert-by-experience (i.e. a service user or carer with personal lived experience of mental health services) to gather participant views of their experience of taking part in the study. If participants agreed to this, they were contacted by an expert-by-experience researcher within 2 weeks to arrange an exit interview. All participants who dropped out of the study after having started on the intervention phase were approached for an exit interview. Research assistants also approach approximately every third participant who completed the study.

**Fig. 1:**
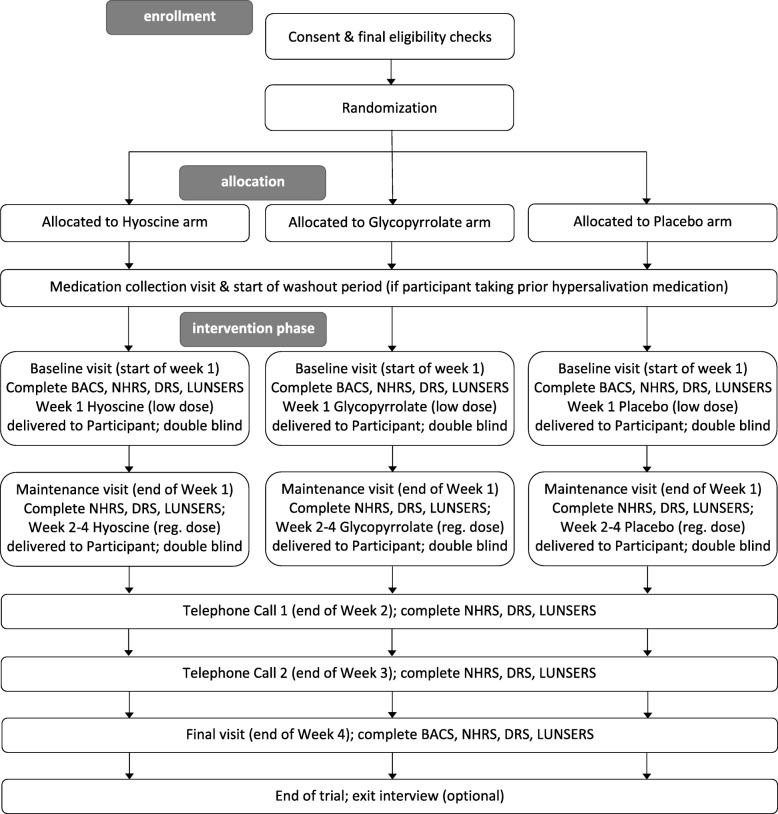
CONSORT diagram showing key stages of study design

The stages of the trial design in each phase are shown in Fig. 1.

### Outcomes

The primary outcome measure was the overall recruitment level and retention rate for the study. The secondary outcome measure was the level of salivation reported using the Drooling Rating Scale, specifically the mean and standard deviation for this measure in the target population.

The criteria for progression to a full randomised controlled trial were set at (1) a recruitment rate at a minimum of one participant per site per month and (2) an overall attrition rate of less than 40%.

### Feasibility measures

The recruitment rate was calculated by dividing the total number of consented participants by the number of active recruiting sites, and the attrition rate was the total proportion of consented participants who left the study before completing the intervention phase. Both indicators were standing items for discussion at all trial management group meetings.

### Study measures

During the study, salivation was measured using the Drooling Rating Scale and the Nocturnal Hypersalivation Rating Scale, cognition was measured using the Brief Assessment of Cognition in Schizophrenia and side effects were measured by the Liverpool University Neuroleptic Side-Effect Rating Scale.

#### The Drooling Rating Scale (DRS)

The DRS [18] is a 2-item scale comprising drooling severity and frequency assessments that combine to form a score ranging from 2 to 9. Whilst it has not been validated and its metrics (standard deviation, mean, sensitivity to change) are unknown in a UK clozapine-induced hypersalivation population, it has good face validity and has been used in published research on paediatric hypersalivation. DRS was used weekly to assess daytime hypersalivation, and establishing its metrics in a UK CIH population was a feasibility aim.

#### The Nocturnal Hypersalivation Rating Scale (NHRS)

The NHRS [22] is a validated single-item 5-point self-report scale for measuring the degree of nocturnal salivation that a respondent experiences. The NHRS is the only scale specifically mentioned in the Cochrane review for treatments for CIH that is recommended for inclusion in future studies of the efficacy of CIH interventions.

#### Brief Assessment of Cognition in Schizophrenia (BACS)

The BACS [23, 24] comprises a short battery of tests devised for easy administration and scoring which assess the extent of cognitive impairment in schizophrenia. The battery includes brief assessments of executive function, verbal fluency, attention, verbal memory, working memory and motor speed and requires approximately 30 min to complete. The BACS has high test–retest reliability in people with schizophrenia and healthy controls and has been shown to be as sensitive as a standard 2.5-h test battery.

#### Liverpool University Neuroleptic Side-Effect Rating Scale (LUNSERS)

LUNSERS [25] is a 51-item checklist of side effects which asks for ratings on a 5-point scale of the degree to which respondents have experienced that side effect in the last month. It can be completed in less than 10 min and shows good reliability and validity, correlating well with other clinician-administered side effect scales. A modified LUNSERS was used to assess side effects in the previous week (rather than month).

#### Exit interviews

A subset of participants also completed an exit interview conducted by an expert-by-experience researcher. Semi-structured interviews explored the participant’s experience of taking part in the study, covering the acceptability of the study methods and soliciting advice on how their study participation experience could have been improved. Exit interviews were requested with all participants who did not complete the study and a selection of completing participants.

### Data security and monitoring

Data entry was completed by the research assistants and validated by the study manager who completed validation checks on a minimum of 10% of the complete participant data. All source data and trial documentation were made available to MAHSC-CTU for study monitoring. Participants consented to this within the consent process.

Participant contact details were disclosed to the research assistants for the purposes of arranging consent and data collection visits. All participant data collected by the research assistant were identified using the participant’s study code and initials. Identifiable participant information was kept in a locked filing cabinet and separate from the participant’s study data.

Study monitoring was conducted by MAHSC-CTU. The Quality Assurance Monitor checked case report forms, trial master file and other trial documentation for completeness and the compliance of the trial team with the protocol and good clinical practice (GCP) standards.

### Trial management group

The trial management group was formed comprising the Chief Investigator, co-investigators, study manager, research assistants, MAHSC-CTU representatives, service user representative, and pharmacy representative. The group met every 2 months.

### Ethical approval and consent

The study was conducted in accordance with the ethical principles prescribed in the Declaration of Helsinki. The patients were given an information sheet for consideration and given a minimum of 2 days to decide on participation. Written informed consent was obtained at a consent interview and patients were given an opportunity to ask questions before completing the consent form. Formal consent was obtained before any study procedures took place.

Ethical approval for the study was granted by the Health Research Authority, North West - Greater Manchester East Research Ethics Committee (reference 15/NW/0823).

### Study sites and data collection

Mersey Care NHS Foundation Trust opened as a study site in January 2017. Mersey Care operates three community clozapine clinics, with approximately 400 registered patients in total, and patients at all three clinics were approached about participation by clinic staff. Lancashire Care NHS Foundation Trust was opened as a study site in February 2017 and eight of the community clozapine clinics, with approximately 600 registered patients, were recruited from. The third study site was not opened due to internal service re-organisation at that site and consequently in August 2017, with the agreement of research funding body, the recruitment target was adjusted from 42 participants across three sites to 28 participants across two sites.

One researcher omitted to enter participant data centrally during data collection and missing data for four participants was reported to the Health Research Authority (HRA) in November 2018 as a protocol breach.

## Analytic strategy

### Quantitative data

No hypothesis testing was conducted as the feasibility aims were to assess the recruitment and retention rates in the target population and establish the metrics of the putative primary outcome measure; the mean and standard deviation for this measure are reported.

### Qualitative data

Exit interviews were conducted with participants after they completed (or dropped out of) the trial in order to gather information on their experience of trial participation and trial procedures. This was to acquire an understanding of how a future trial could be designed and common themes are reported.

## Results

### Recruitment and retention

**Fig. 2:**
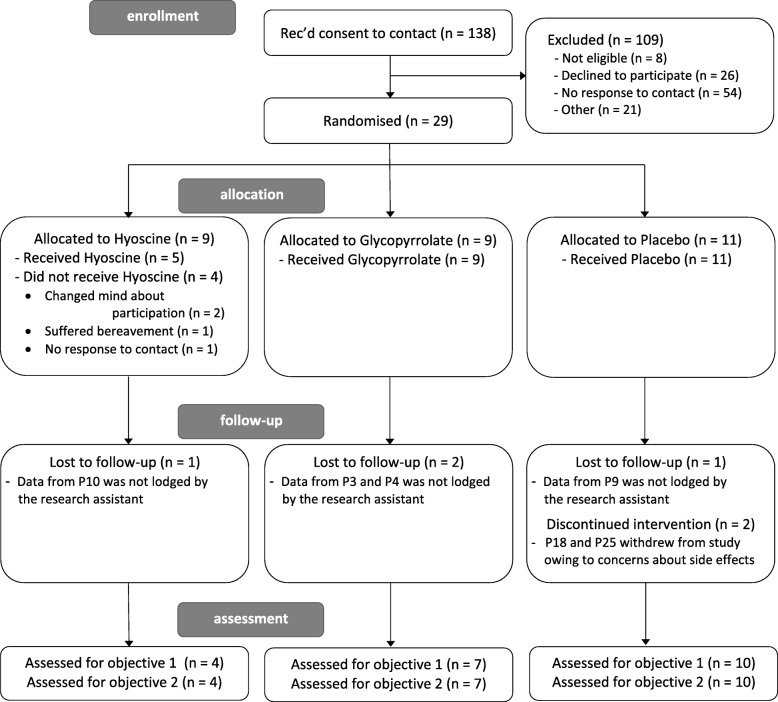
CONSORT diagram showing the number of patients who consented to contact, were randomised, were followed-up and complete datasets analysed

The number of patients assessed for eligibility, randomised and excluded, and the number of complete datasets assessed in each trial arm are presented in the CONSORT diagram in Fig. 2.

A total of 138 patients consented to being contacted by a researcher and 29 participants were randomised to the study; this represents 21% of participants that agreed to be contacted and Table 1 shows the demographic details of the sample. Nine participants were randomised to the hyoscine arm, nine to the glycopyrrolate arm and 11 to the placebo arm. However, four participants (all from the hyoscine arm) exited the study before starting the intervention phase of the trial: two participants changed their minds and gave no further reason for leaving the study; one participant left due to a change in personal circumstances; one participant did not respond to contact after having been randomised and was withdrawn after 6 weeks of no contact. Eighty-two percent of randomised participants therefore received study medications.

**Table 1:** Age and gender distribution in each arm at time of randomisation

Arm	Age in years	Frequency (gender)
Hyoscine	25–34	0
	35–44	5 (2 female, 3 male)
	45–54	2 (2 male)
	55–64	2 (2 male)
Glycopyrrolate	25–34	1 (1 male)
	35–44	4 (4 male)
	45–54	2 (1 female, 1 male)
	55–64	2 (2 male)
Placebo	25–34	3 (1 female, 2 male)
	35–44	4 (1 female 3 male)
	45–54	2 (2 male)
	55–64	2 (2 male)

In terms of acceptability of the study design, using telephone interviews to collect data, we established this was both acceptable to participants and viable in practice.

### Tolerability of the study drugs

Of the 24 participants who received study medication only two participants exited during the study intervention phase. Both these participants were in the placebo arm; one participant perceived their hypersalivation to be worsening and one participant left the trial because their blood tests, conducted as part of normal clozapine treatment monitoring, showed abnormal results and although this was unlikely to be linked to the trial medication, decided to leave the trial. Nevertheless, data from these participants were still included in assessing the trial objectives as they were able to provide measures of salivation using the DRS during the baseline phase and were able to contribute data to the assessment of recruitment and attrition.

There were no dropouts from either the hyoscine or glycopyrrolate arms demonstrating these medications were well tolerated and no serious adverse events were reported during the trial. There was one report of a medication error; a participant was prescribed medication for hypersalivation prior to being recruited to the trial and an error resulted in their previous hypersalivation medication being prescribed concurrently with trial medication. The error was noticed after 3 days and trial medication was stopped immediately. The participant re-joined the trial later after the previous medication was confirmed to have been removed. This event led the research team to improve the procedure on confirming that relevant prescribed medication had been discontinued.

In summary, our recruitment and retention aims were met as 29 participants were recruited that met the revised recruitment target demonstrating the trial design was acceptable. Study participants found the medications to be tolerable and the attrition rate was 24% which was significantly less than the 40% attrition rate identified a priori as the progression criterion.

### Parameters of primary outcome measure

A secondary aim of the trial was to establish the baseline metrics of the DRS. Twenty-one participants (19 participants who completed the trial and 2 participants who dropped out after receiving trial medication) provided hypersalivation data using the DRS when baseline measurements were recorded. The mean DRS score at baseline was 5.14 and the standard deviation was 1.68.

### Exit interviews

A service user researcher, accompanied by a researcher, conducted seven exit interviews with participants. Six interviews were conducted with participants who completed the trial and one interview with a participant who withdrew from the study owing to worsening side effects (excessive salivation). The primary aim of the exit interviews was to assess the participants’ experiences of trial participation and to obtain suggestions about changes that may have improved their experience.

Two main themes emerged from our analysis of the exit interviews namely that consent was well informed and trial procedures were acceptable. The themes together with illustrating quotations are presented in Table 2. The recruitment method, washout period, trial procedures and data collection were all reported as acceptable, and no respondents raised any difficulties with any of the trial processes. Without exception, all interviewees expressed very positive views of trial participation. Data from exit interviews also suggested that participants were reassured by the presence of a researcher during recruitment who was able to answer questions about the trial (see quotations from P13 and P14 in Table 2) and that this was an important aspect of successful recruitment. This aspect of the recruitment strategy is something that we aim to strengthen in the future efficacy trial by having a researcher present at the initial approach to potential participants.

**Table 2:** Main themes with illustrative quotations from the exit interviews with 7 participants

Recruitment was well informed	
“I did have some questions about effectiveness and side effects but I was ok with the responses from the [clinic nurses]. It might have been better if you [the researcher] were in the room when I was first asked so you could answer my questions – I think it’s better to have someone there the first time people are asked about it.” [P13]	
“I felt like it was quite thorough.” [P25]	
“I was nervous at first and had some questions about side effects and the nurses did not really know but when I spoke to [the researcher], she was able to answer these and shewas reassuring that I did not have to take part and if I did I could stop whenever. But yeahI had to wait to speak [the researcher] and it would be better to have those questionsanswered there and then.” [P14]	
“Yeah I knew everything I needed to know.” [P06]	
Trial procedures were acceptable	
“The cognitive test thing was fairly interesting to do – it was something different.” [P13]	
“The tests were fun in a way. 45 minutes is a long time but actually it was fun. I did not realise the time had gone by.” [P14]	
“I think it went really well. The meetings and phone calls were not too much.” [P23]	
“I think it went alright. I cannot see any improvement on it. I would not change anything.” [P23]	

## Discussion

The trial presented several challenges in an under-researched population and has provided important information for the design and conduct of a future RCT. A major aim of the feasibility study was to assess recruitment strategies and recruitment rates and explore how these could be improved for future studies. We found our trial design to be acceptable, and 138 patients agreed to receive further information about the trial of which 29 were eventually randomised. Across two sites and 11-months, this equated to 1.3 participants per site per month, which comfortably exceeded our a priori progression criterion for recruitment rate of 1 participant per site per month. The presence of a researcher in the clozapine clinics during recruitment, who could explain the trial in detail and answer questions, proved to be an important factor in improving recruitment. Valuable lessons were also learned liaising and maintaining relationships with clozapine clinic staff making the initial approaches to patients and promoting the trial to patients using posters before they were approached.

Our results also confirmed that participants found the design to be acceptable and medications to be tolerable; the attrition rate was 24% which is comparable to that found in other trials involving patients with clozapine-induced hypersalivation [10] and was lower than the 40% attrition rate identified a priori as a criterion for progression. Participants were in weekly contact with a researcher throughout the intervention phase, and it is likely that this level of involvement and engagement with the research team contributed to low levels of attrition.

Of the 138 patients who agreed to be contacted by a researcher about participation in the study, 29 ultimately consented to participation. Of the remaining 109 patients, 8 were found to be ineligible, 26 declined to take part in the study after hearing more details about it, and 21 could not take part for other miscellaneous circumstances (e.g. could not begin study participation before the end of study date, going on holiday). Fifty-four patients could not be contacted to follow-up on their initial expression of interest, and in these cases, the patient left an address and telephone number as their contact details but later failed to answer numerous attempts at telephone contact by the researcher. After a week, the patient was sent a letter thanking them for their interest in hearing more about the study, and asking them to telephone the research office if they would like to hear more. It was expected that it would be difficult for some patients to respond to contact and answer a telephone call (particularly from an unknown caller). It is also possible that patients may initially have agreed to hear more about the study when approached by clinic staff but later felt less motivation to follow through and respond to a letter. It has already been noted that recruitment rates were higher when a researcher was present in the clinic when the patient agreed to hear more about the study and one reason for this may be that the patient had the opportunity to learn more about the study whilst they were still motivated and receptive to participation. Nevertheless, the target population—community patients with a diagnosis of treatment-resistant schizophrenia—are a hard-to-recruit population and recruitment difficulties were expected. Future studies that aim to recruit from this population should aim to provide further information about the study as soon as possible after an initial expression of interest from the patient.

Owing to the opening of only two study sites rather than three, the recruitment target was reduced proportionally and the revised target was met. However, the smaller sample means that estimates of the key parameter in the primary outcome measure (the Drooling Rating Scale) is likely to be a more imprecise estimate than would have resulted from a larger sample. A smaller sample size also means that the probability of detecting adverse events is reduced.

Nevertheless, the trial was able to provide informative data that met the trial aims; recruitment and retention rates were successfully estimated, and metrics of the primary outcome measure were obtained to inform a future sample size calculation. The research team gathered valuable experience of recruiting participants from community clozapine clinics and appreciated the importance of effective communication with clinic staff and patients, as well as engaging patients and informal caregivers in the communication and promotion of research.

Our conclusion is that a large, multi-centre RCT using a placebo arm and telephone interviews is feasible in a trial assessing the effectiveness of medications for clozapine-induced hypersalivation.

## Data Availability

The datasets used and/or analysed during the current study are available from the corresponding author on reasonable request.

## Abbreviations

BACS: Brief Assessment of Cognition in Schizophrenia
CIH: Clozapine-induced hypersalivation
CONSORT: Consolidated Standards of Reporting Trials
DRS: Drooling Rating Scale
GCP: Good Clinical Practice
IMP: Investigational medicinal product
LUNSERS: Liverpool University Neuroleptic Side-Effect Rating Scale
MAHSC-CTU: Manchester Academic Health Sciences Centre-Clinical Trials Unit
NHRS: Nocturnal Hypersalivation Rating Scale
RCT: Randomised controlled trial
